# The utilisation of legal instruments by United Nations actors to restrict the exposure of children to unhealthy food and beverage marketing: a qualitative content analysis of UN instruments

**DOI:** 10.1186/s12992-023-00939-4

**Published:** 2023-06-30

**Authors:** Fiona Sing, Sally Mackay, Margherita Cinà, Boyd Swinburn

**Affiliations:** 1 School of Population Health, University of Auckland, Auckland, 1023 New Zealand; 2 O’Neill Institute for National and Global Health Law, Georgetown University Law Center, Washington, US

**Keywords:** Global health law, Marketing, Child rights, Global health governance

## Abstract

**Introduction:**

United Nations (UN) agencies are influential global health actors that can introduce legal instruments to call on Member States to act on pressing issues. This paper examines the deployment and strength of global health law instruments used by UN actors to call on Member States to restrict the exposure of children to unhealthy food and beverage marketing.

**Methods:**

Global health law instruments were identified from a review of four UN agencies that have a mandate over children’s exposure to marketing of unhealthy food and beverage products namely: the World Health Organization (WHO); the Food and Agriculture Organization (FAO); the United Nations General Assembly (UNGA) and the UN Office of the High Commissioner for Human Rights (OHCHR). Data on marketing restrictions were extracted and coded and descriptive qualitative content analysis was used to assess the strength of the instruments.

**Results:**

A wide range of instruments have been used by the four agencies: seven by the WHO; two by the FAO; three by the UNGA; and eight by the UN human rights infrastructure. The UN human rights instruments used strong, consistent language and called for government regulations to be enacted in a directive manner. In contrast, the language calling for action by the WHO, FAO and UNGA was weaker, inconsistent, did not get stronger over time and varied according to the type of instrument used.

**Conclusion:**

This study suggests that a child rights-based approach to restricting unhealthy food and beverage marketing to children would be supported by strong human rights legal instruments and would allow for more directive recommendations to Member States than is currently provided by WHO, FAO and UNGA. Strengthening the directives in the instruments to clarify Member States’ obligations using both WHO and child rights mandates would increase the utility of global health law and UN actors’ influence.

**Supplementary Information:**

The online version contains supplementary material available at 10.1186/s12992-023-00939-4.

## Introduction

Children are exposed to persistent and high volumes of unhealthy food and beverage marketing, [1, 2] creating social norms and increasing preference and consumption of these foods [2–6]. This food marketing goes beyond traditional advertising, such as on broadcast media, and includes point of sale techniques, product packaging, brand marketing, and sponsorship. This marketing phenomenon is driving increased consumption of unhealthy food and beverages contributing to a shift in diets towards ultra-processed foods [7]. Across the life course, this contributes to an increased risk of overweight and obesity, cognitive impairments, reduced quality of life and non-communicable diseases (NCDs) [7, 8]. The prevalence of childhood overweight and obesity globally is already at unacceptable levels with 38.2 million children under 5 years of age with overweight or obesity as at 2019 and over 340 million children and adolescents aged 5–19 with overweight or obese as at 2016 [8–10]. Such predatory marketing techniques, and the consequential effects, not only impact on children’s physical and mental health, but they are also considered a breach of a child’s right to health under the United Nations Convention on the Rights of the Child (UNCRC) [11, 12].

UN agencies play an important role in global health governance due to the wide range of areas that health intersects with as well as the wide reaching scope of the various mandates of the different structures within the UN system. UN agencies can monitor and communicate the extent of the problem, such as obesity rates or human rights issues; they can provide technical guidance and capacity building to help Member States address those issues; and they can use global health law instruments, such as conventions or principles and standards, to influence Member States to act [13]. UN agencies hold a position of power in the global health governance system to address the issue of unhealthy food marketing and its impact on child health and child rights. This paper focuses on the utilisation of global health law instruments by relevant UN actors to call on Member States to address the exposure of children to unhealthy food and beverage marketing.

The rapidly evolving global health environment, largely caused by globalisation, has necessitated the emergence of global health law, an area of law that addresses the complex and dynamic nature of global health [12, 13]. It emerges from the more traditional field of international health law, which originally focused on the relationships between governments, in particular as they related to the international spread of specific infectious diseases. With the rise of globalisation and our interconnected world, global health law became the predominant terminology, encompassing a wide range of new actors including multilateral organizations like the UN, and a corresponding range of instruments relevant to this growing field [14–16].

There is a range of global health law instruments UN actors can utilise to call on Member States to act in relation to unhealthy food marketing [13, 14, 17]. Scholars define this suite of documents as encompassing a scale ranging from non-binding voluntary formal norms (no obligation to uphold the norms) backed by an authoritative body such as the International Code of Marketing of Breastmilk Substitutes to binding formal norms negotiated by authoritative stakeholders (i.e. governments) such as the WHO International Health Regulations (2005) or the Framework Convention on Tobacco Control [17, 18]. Gostin et al. frame these as ‘soft’ and ‘hard’ laws [14, 18] with soft laws constituting international instruments, other than treaties, that contain non-binding principles, norms, standards, or other statements of expected behaviour [19]. In international law, hard law (e.g. treaties and conventions) creates legally binding obligations on parties involved, which can be enforceable through relevant international institutions.

While non-binding norms may not appear as formal in a legal sense in domestic law, scholars argue that their normative weight and the influence they can have on actor behaviour, in particular Member States, in terms of domestic law and policy, means it is important to take a broad view of the definition of global health law that encompasses these instruments [17, 18]. Gostin observes that hard and soft legal instruments are often similar in form as both are negotiated and adopted by Member States, are usually administered by international organizations like the UN, and can be enforced using similar mechanisms [18]. For example, the WHO International Code of Marketing of Breast Milk Substitutes is not a binding international law, but over time its normative strength has increased, so that it holds a similar authoritative weight to harder laws. The Code is therefore an authoritative document that holds normative weight and influence, and can be used to hold governments accountable for their obligations around the marketing of breast milk substitutes.

In relation to unhealthy food and beverage marketing, four UN agencies have overlapping mandates: the World Health Organization (WHO), the Food and Agriculture Organisation (FAO), the UN General Assembly (UNGA) and the UN Office of the High Commissioner for Human Rights (OHCHR). All four use various instruments within their jurisdiction to comment on the issue of children’s exposure to unhealthy food and beverage marketing. Each entity has a different rationale for addressing unhealthy food marketing, for example reducing non-communicable diseases (WHO), reducing malnutrition in all its forms (FAO and WHO) or protecting children’s right to the highest attainable standard of health (Human Rights agencies), and different mechanisms by which they develop their instruments.

A mapping and analysis of the different global health law instruments utilised by multiple UN actors has not been undertaken in relation to food marketing before. Understanding the strengths and weaknesses of the current global health law environment in relation to unhealthy food and beverage marketing can inform a further discussion about the utility of global health law to address the global health issue. A comparison of the instruments of all four agencies provides a deeper analysis of the global health law landscape, over time, and its likely impact on Member States. Assessing the strength and nature of the instruments may help explain the global policy inertia in regulating the ultra-processed food and beverage industry and illustrate how these instruments could be strengthened to improve Member State’s actions. Further, understanding how the different mandates of the UN agencies overlap and potentially work in unison is also an important study as the UN agencies begin to increase their multisectoral and coordinated responses on global health and food system issues.

While each entity has different mandates, they also have different tools and instruments at their disposal to create obligations of varying strengths. This paper seeks to examine the normative instruments and legal levers used by each of these UN actors to call on Member States to restrict the exposure of children to unhealthy food and beverage marketing. The paper first maps out the various instruments used by those agencies, then analyses the nature and content of those instruments to understand the strength of the instruments by assessing the language used.

## Methods

The methods for this study were designed to answer the research question - how have UN agencies called on its Member States to restrict the exposure of children to unhealthy food and beverage marketing over time? As the intended focus of the research was global health law, the study looked at how the UN agencies have used their formal powers to call on Member States to act through a structured search for UN instruments to identify the key documents followed by a qualitative content analysis of those documents.

### Data collection

A structured search was undertaken to identify key instruments from each of the four UN agencies: the WHO; the FAO; UNGA; and the OHCHR. A search was undertaken of the relevant UN agencies’ websites, key UN reports that reference UN instruments and peer-reviewed journal articles discussing the topic of marketing to children to collate the list. The details regarding the search methods are outlined in Online Supplementary Material 1.

The inclusion criteria were that: firstly, the document was a UN level agency instrument that called on Member States to act in some way to reduce children’s exposure to unhealthy food and beverage marketing, as opposed to technical advice to aid Member States such as WHO reports on policy design. Secondly, to qualify as an ‘instrument’, the document had to be a normative or legal global health law instrument that fit under the governance structures of that entity. A definition of ‘instrument’ was adopted from Moon’s work as ‘a codified rule (whether binding or non-binding) with the explicitly-stated intention to protect or promote health, endorsed by a governmental or intergovernmental entity, agreed by three or more countries and with effects beyond a single region’ [17]. Therefore, thirdly, the instrument had to be relevant to all Member States, not specific regions. This third criterion was also chosen because the focus of the study was global, so instruments had to address all Member States. While it is accepted that regional instruments could also influence Member States to act, the scope of this study was confined to global instruments in the first instance so that the corpus of data was manageable.

**Fig. 1:**
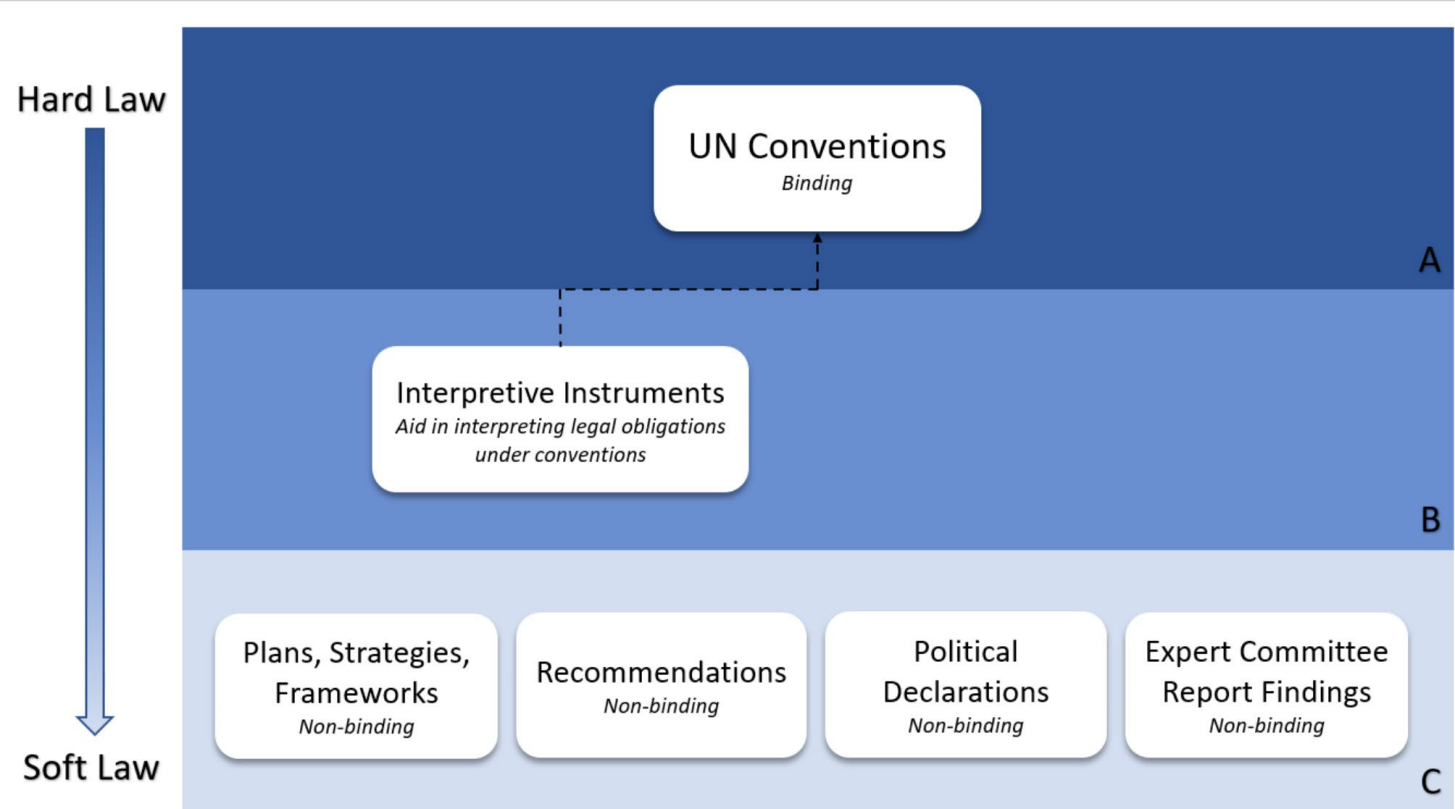
A hierarchy of relevant global health law instruments Under the WHO Convention, the WHO has the ability to introduce binding regulations, but only for specific health topics like infectious disease prevention, none of which relate to preventing non-communicable diseases. WHO Regulations are therefore not indicated in Fig. 1. While the instruments in category C are all different in their construction, legally they have the same standing as non-binding instruments [13]

Figure 1 outlines a hierarchy of relevant global health law instruments that each entity has the jurisdiction to use. The instruments range from ‘hard laws’ in Row A through to ‘soft laws’ in Row C with interpretive instruments in the middle which are instruments that support ‘hard laws’ and are to be read as part of those instruments. Table 1 also illustrates what each of these categories mean.

### Data analysis

After the instruments were identified, the key attributes of the instrument were extracted into an Excel document, namely: the date; the official name of the document; the UN entity responsible; the type of instrument (Convention, Code, General Comment, Special Rapporteur Report, Plan, Strategy, Recommendation, Declaration for example); and any reference in the substantive content (i.e. the wording of the text) of the instrument relating to unhealthy food and beverage marketing. All UN instruments were available in English so language was not a barrier to the authors.

A qualitative content analysis was then carried out to assess the strength of the instruments. The authors considered that the strength of the instrument could be characterised by two dimensions: the type of instrument (see Table 1) and the language used in the substantive content of the instrument. An analysis of these two elements (the type of instrument and the strength of the instrument) could discern where the instrument sat on the spectrum of legal instruments, in terms of whether it was binding or non-binding for example; and how strong the language calling on Member States to act was.

Table 1 outlines the description of types of instruments categorised into three categories A, B and C.

**Table 1:** Description of global health law instruments

Category A	Binding instruments: binding formal norms negotiated by authoritative stakeholders (i.e. governments). Examples: UN Convention on the Rights of the Child, Framework Convention on Tobacco Control	Hard Law
Category B	Interpretive instruments: instruments used to interpret what the treaty means – considered part of the treaty law. Examples: (Committee on the Rights of the Child General Comment No. 15 (2013) on the right of the child to the enjoyment of the highest attainable standard of health)	Soft law
Category C	Non-binding instruments: voluntary formal norms backed by an authoritative body that are not binding (no obligation to uphold the norms). Examples: WHO Global Strategies, Action Plans or Frameworks for Actions. Codes such as the International Code of the Marketing of Breastmilk Substitutes	

To analyse the language in the global health law instruments, the authors drew on a well-established policy content analysis tool (WellCCat) developed by the Rudd Centre and adapted for use in other studies, including one of the INFORMAS network’s modules [20–24]. The attributes adopted from the policy analysis tool focused on the strength of language of the statements being communicated from one policy entity to another policy entity regarding policy action. Statements considered to be ‘weak’ included those that would be hard to enforce as they are vague or unclear; focused on goals, suggestions, objectives or recommendations; and utilised language such as may, can, could, should, might, encourage, suggest, and urge. In contrast, stronger statements would include language that indicates that action or regulation is required, utilising words such as shall, will, must, have to, insist, require, comply and enforce.

To assess the strength of the instrument, the authors selected a benchmark considered to be the ‘strongest’ directive from UN agencies to Member States to act, which was a legally binding document (category A) that used strong language to call on Member States to introduce a mandatory regulatory response. This benchmark was chosen as consistent independent evaluations assessing the effectiveness of unhealthy marketing restrictions globally have shown that mandatory approaches are more effective at reducing the power and exposure of unhealthy food marketing [25–29]. On the other hand, government-led or industry-led voluntary codes are, more often than not, found to be ineffective [25, 30–36]. Given this, any explicit mention in the instrument calling on Member States to work with or consult with the food and beverage industry was by proxy considered a weak directive.

With this conceptual underpinning, a content analysis was undertaken assessing the following questions:

- What action words are used – how are Member States called on to act?
- In what context are the action words used?
- How does the language change over time across instruments?

## Results

### Mapping of instruments

After mapping the instruments a list of the instruments employed was created. Table 2 outlines the key instruments used in date order.

**Table 2:** List of the key instruments

**WHO**	**2004**- Global Strategy on Diet, Physical Activity and Health [37] **2008**- Action Plan for the Global Strategy for the Prevention and Control of Non-communicable diseases 2008–2013 [38] **2010**- WHO Set of Recommendations on the Marketing of Foods and Non-alcoholic Beverages to Children (**WHO Set of Recommendations**) [39] **2012**- Framework for implementing the WHO Set of Recommendations [40] **2013**- Global Action Plan for the Prevention and Control of Noncommunicable Diseases 2013–2020 [41] **2016**- Report of the Commission on Ending Childhood Obesity [7] **2017**- Appendix 3 of Global Action Plan: Tackling NCDs: ‘Best Buys’ and other recommended interventions for the prevention and control of non-communicable diseases [42].	**Cat C**
**FAO (in collaboration with WHO)**	**2014-** Rome Declaration on Nutrition: Conference Outcome Document of the Second International Conference on Nutrition [43] **2014-** Framework for Action to implement the Rome Declaration on Nutrition [44]	**Cat C**
**UNGA**	**2011**- Political Declaration of the High-Level Meeting of the General Assembly on Non-Communicable Diseases [45] **2014**- Outcome document of the High-Level Meeting of the General Assembly on Non-communicable diseases [46] **2018**- Political declaration of the 3rd High-Level Meeting of the General Assembly on Non-Communicable Diseases [47]	**Cat C**
**UN Human Rights**	**2000-** UN Convention on Rights of the Child (**UNCRC**) [48] **2013-** Committee on the Rights of the Child General Comment No. 15 (2013) on the right of the child to the enjoyment of the highest attainable standard of health (art. 24) [49] **2013-** Committee on the Rights of the Child General Comment No. 16 (2013) on State obligations regarding the impact of the business sector on children’s rights [50] **2014**- Report of the Special Rapporteur on the Right to Health: unhealthy foods, non-communicable diseases and the right to health [51] **2014 -** Report of the Special Rapporteur on the Right to Food: The transformative potential of the right to food [52] **2016-** Report of the Special Rapporteur on the Right to Health: Sport and healthy lifestyles as contributing factors to the Right to health [53] **2020**: Statement by the UN Special Rapporteur on the right to health on the adoption of front-of-package warning labelling to tackle NCDs [54] **2021-** Committee on the Rights of the Child General Comment No. 25 (2021) on children’s rights in relation to the digital environment [55]	**Cat A for UNCRC** **Cat B for remainder**

**Fig. 2:**
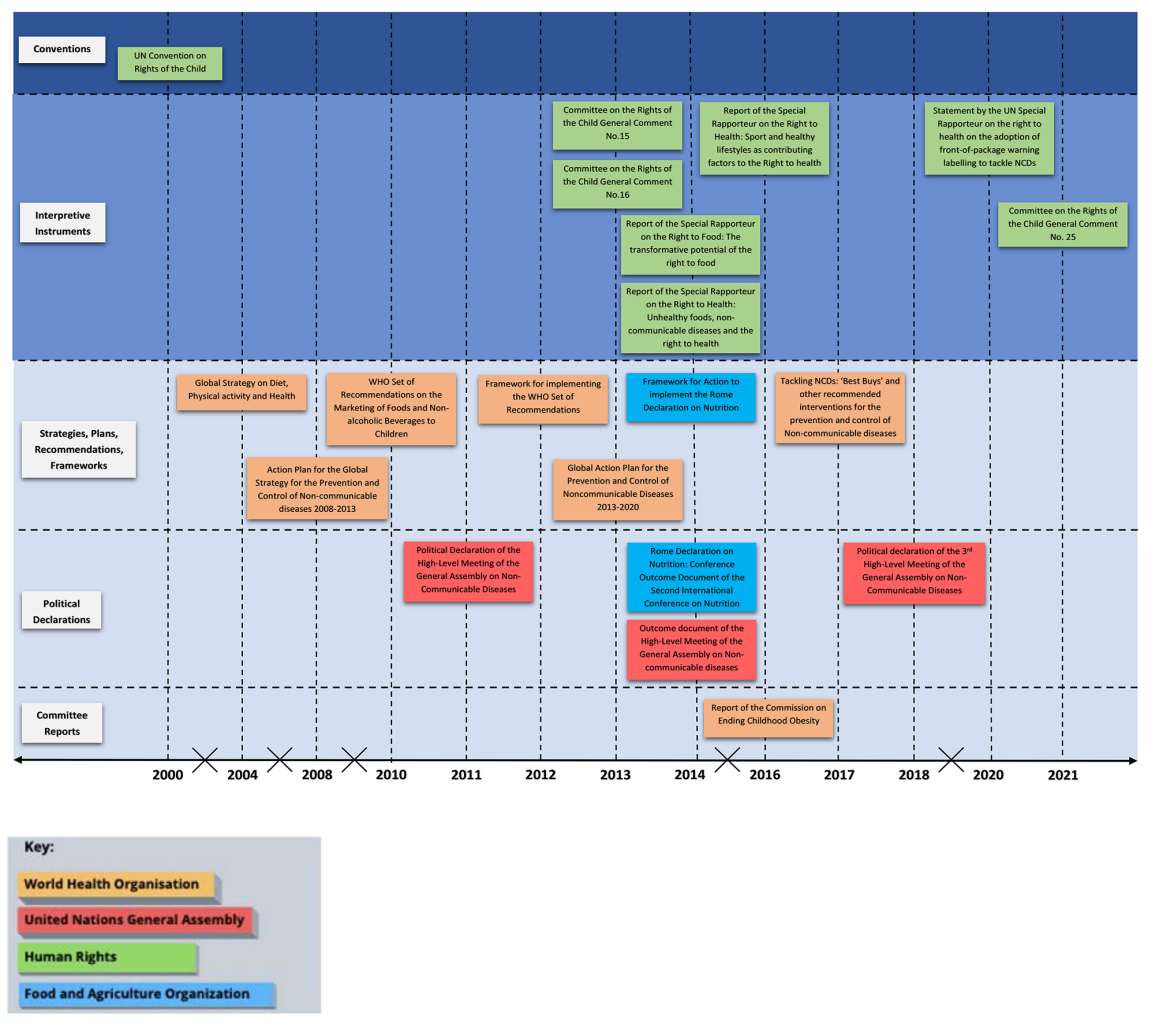
Timeline of global health law instruments introduced 2000–2021

Figure 2 outlines the timeline of the instruments to show the history of the normative and legal levers utilised by each entity studied. The instruments are split into category A,B and C from Table 1 along the y axis. The instruments are then colour coded according to the key to show which UN agency they belong to.

### Nature of instrument and strength of language

#### WHO instruments

Seven WHO instruments spanning thirteen years (2004 to 2017) were assessed. All the instruments fitted into Category C with the WHO Set of Recommendations being the centrepiece that was specific to food marketing [36], [37], [38], [39], [40], [7], [41]. Over time the types of instruments utilised varied, illustrating an ongoing commitment to including marketing restrictions in the global health discourse.

Before 2010, marketing restrictions were mentioned in the Global Strategy (2004) [37] and Action Plan (2008) [38]. The discourse in the WHO instruments began to identify marketing of unhealthy food as an issue, particularly the influence marketing has on food choices and dietary habits. Governments were encouraged to act, but there was a conciliatory approach for governments to work with consumer groups and the private sector - which included the advertising sector - to develop appropriate multi-sectoral approaches to deal with marketing of food to children, and to deal with ‘such issues as sponsorship, promotion and advertising’ [37]. By 2008, the discourse changed, as Member States were encouraged to develop frameworks or mechanisms, to promote ‘responsible marketing’ as opposed to restricting the food and beverage industries more harmful practices [38].

The WHO Set of Recommendations, introduced in 2010, was important, as while it was not a binding document, it was primarily focused on providing Member States with a clear mandate to act on food marketing to children, stating the ‘evidence shows a clear rationale for action’ by Member States [39]. The WHO Set of Recommendations were developed in consultation with Member States and they recognised that Member States acknowledged the need to ‘develop appropriate policy mechanisms’. The main purpose of the WHO Set of Recommendations was stated as ‘to guide efforts by Member States in designing new policies, or strengthening existing policies, on food marketing communications to children in order to reduce the impact of marketing foods high in saturated fats, trans-fatty acids, free sugars or salt’ [39].

The WHO Set of Recommendations called on Member States to choose a policy approach which could range from statutory regulation (government introducing legislation to compel industry to act such as in Chile), to co-regulation (government co-designing the regulatory approach that is then voluntary to adhere to such as the previous regulatory approach in the United Kingdom), to industry self-regulation (industry creates its own policy and voluntarily chooses whether to adopt it or not such as the EU Pledge). While the WHO Set of Recommendations did not stipulate that a statutory regulation was the most appropriate option, which would have been the strongest directive, it did recommend that ‘governments were in the best position to set direction and overall strategy’ and that when governments engaged with other stakeholders ‘care should be taken to protect the public interest and avoid conflict of interest’. Communication with all stakeholder groups was encouraged. When Member States endorsed the WHO Set of Recommendations with World Health Assembly Resolution 63.14, the resolution urged Member States ‘to take necessary measures to implement the recommendations’; identify the most suitable policy option given national circumstances; and to cooperate with civil society, public and private stakeholders in implementing the recommendations ‘while ensuring avoidance of potential conflicts of interest’ [42]. However, including such a wide range of regulatory options weakened the directive overall.

Following its publication, all further WHO instruments referred to Member States implementing the WHO Set of Recommendations, including the Global Action Plan on the Prevention and Control of NCDs 2013–2020 [41]; the WHO Commission on Ending Childhood Obesity [7]; as well as the WHO ‘Best Buys’ and other Recommended Interventions for the Prevention and Control of NCDs [43]. The language ranged from a weaker directive of ‘consider implementation of’ in the WHO Set of Recommendations [41] to a stronger directive of ‘urging Member States’ [7] and give ‘high priority’ to the implementation of the WHO Set of Recommendations ‘as being integral to making progress towards the voluntary global targets’ [7].

The WHO Commission on Ending Childhood Obesity (ECHO Commission), made up of 15 independent expert commissioners, who were selected because of their eminent positions in the field, provided a further directive on the lack of appropriate action by Member States, stating that the exposure of children to unhealthy food and beverage marketing was still a ‘major issue’ ‘demanding change’ [7]. The Commission report expressly voiced its position that Member States had failed to ‘give significant attention’ to the WHA63.14 Resolution that implemented the WHO Set of Recommendations and called for this issue to be addressed by Member States by ‘developing regulations in line with the WHO Set of Recommendations’ [7].

In 2017, the WHO published ‘Tackling NCDs: ‘Best Buys’ and other Recommended Interventions for the Prevention and Control of NCDs’ that listed a set of policy interventions Member States could introduce to address NCDs. ‘Implementing the Set of Recommendations’ was omitted from the ‘Best Buys’ i.e. the most favourable policy options but it was listed as an ‘overarching/enabling action’ [43]. The status of an overarching/enabling action opposed to a Best Buy policy intervention was not clear, but the omission could have been due to a lack of data available to ascertain how cost effective the policy intervention would be, or it could be a political decision not to prioritise marketing restrictions. But the message this distinction could send Member States regarding the lack of importance of the WHO Set of Recommendations is important to note because it could weaken the directive from the WHO to Member States to implement the WHO Set of Recommendations.

The WHO Set of Recommendations, is an instrument that sets a benchmark for how Member States should address the issue of food marketing. However, this benchmark document is an example of a soft law and while the instrument is dedicated to directing Member States to act on the issue, it fails to propose a mandatory government-led response to the issue. What is then important to note is that subsequent instruments also adopted language directing Member States to act regarding that instrument, which varied over time. The ECHO Report provided the strongest directive to Member States and was the most critical of the performance of those States to implement the WHO Set of Recommendations. This contrasts with the ‘Best Buys’ instrument, introduced one year later, that did not direct Member States to implement marketing restrictions in the same way as other policies. Therefore, the language did not strengthen over time.

#### FAO instruments

Two FAO instruments introduced in 2014 were assessed, both of which fit into Category C [43, 44]. The Rome Declaration on Nutrition [44] was an outcome document of a large inter-governmental conference on Nutrition held in 2014. The Framework for Action [45] was drafted to aid Member States to implement the Rome Declaration.

The language in the Rome Declaration was less direct, stating that inappropriate marketing of food and non-alcoholic beverages to children should be avoided ‘as recommended by resolution WHA63.14’ (the resolution endorsing the WHO Set of Recommendations). The Declaration also recognizes that ‘governments should protect [...] children, from inappropriate marketing and publicity of food’ [44]. However, a specific recommendation in the Framework for Action to implement the Rome Declaration was that Member States ‘regulate’ the marketing of food and non-alcoholic beverages to children ‘in accordance with the WHO Set of Recommendations’. This was the first use of the word regulate (and was later used in the ECHO Report), although this could cover a host of regulatory options not just mandatory government legislation [45].

#### UNGA instruments

As a result of the three High Level Meetings on NCDs held in 2011, 2014 and 2018, a set of commitments were produced, [45, 46] all Category C instruments. In 2011 and 2014, the WHO Set of Recommendations were discussed. In 2011, the Political Declaration ‘promotes the implementation of the WHO Set of Recommendations’. The Declaration states that the parties (Member States) agree to ‘promote the development and initiate the implementation, as appropriate, of cost-effective interventions to reduce high fat, sugar and salt products including through discouraging the marketing of foods that contribute to unhealthy diets’ [47].

In 2014 the Outcome Document called for a mobilization of political will and financial resources to restrict marketing and advertising to children. The Document stated it ‘recognised that the implementation of the WHO Set of Recommendations will accelerate efforts to reduce non-communicable diseases’ [46].

However, at the third and most recent High-Level Meeting in 2018, the Political Declaration was silent on the WHO Set of Recommendations. Instead, the only mention of marketing restrictions is in reference to inviting the private sector to commit to further reduce the exposure of children to unhealthy food and beverage marketing [48].

Three instruments spanning five years were assessed. The language directing Member States to act on food marketing weakens significantly over time. It is unclear from the documents what the cause of this weakening is but options include political pushback, emerging evidence of lack of effectiveness of policies, or increasing uncertainty about the optimal action.

#### UN human rights instruments

Eight instruments spanning twenty-one years (2000 to 2021) were analysed: one overarching convention - the UNCRC (Category A) [49] - and seven instruments that aid in interpreting the obligations of Member States under that Convention (Category B) [49], [50], [51], [52], [53], [54], [55].

The instruments and the mechanisms for action addressing food marketing are different in the UN human rights field. The UNCRC is the overarching instrument used which codifies, in Article 24, the duty of Member States to respect, protect and fulfil the child’s right to the highest attainable standard of health and in Article 6 that Member States will ensure the survival and development of the child [56]. To understand how to implement those Articles, other UN instruments such as the General Comments from the Committee on the Rights of the Child, the Reports of the Special Rapporteurs and the Country Reports from the Committee aid in interpreting the UNCRC and provide direction to Member States [56].

In 2013, the Committee on the Rights of the Child provided a General Comment on Article 24 stating that the marketing of foods and drinks high in fat, salt or sugar ‘especially when such marketing is focused on children – should be regulated’ [50]. In General Comment on Business and Children’s Rights No 16, the Committee stated that ‘preventative measures such as effective regulation and monitoring of advertising and marketing’ will be necessary to implement Article 6 [51].

In 2021, a General Comment regarding children’s rights in the digital environment was issued. This was a call for stronger regulation of the digital environment, and in particular reference to unhealthy food and beverage marketing. The instrument states that all targeted or age-inappropriate advertising, marketing and other digital services ‘should be regulated [...] to prevent children’s exposure to certain food and beverages’ (among other things) [57].

Three Special Rapporteurs have included directions to Member States in their annual reports to the Human Rights Council. While these reports are not solely concerned with the UNCRC, they still provide guidance on the topic of marketing to children. In 2014, a Special Rapporteur made the most compelling directive to Member States:

“*Owing to the inherent problems associated with self-regulation and public–private partnerships, there is a need for States to adopt laws that prevent companies from using insidious marketing strategies. The responsibility to protect the enjoyment of the right to health warrants State intervention in situations when third parties, such as food companies, use their position to influence dietary habits by directly or indirectly encouraging unhealthy diets, which negatively affect people’s health. Therefore, States have a positive duty to regulate unhealthy food advertising and the promotion strategies of food companies. Under the right to health, States are especially required to protect vulnerable groups such as children from violations of their right to health.*” [51]

The Special Rapporteur on the Right to Food stated in a report in 2014 that to reshape food systems Member States should ‘adopt statutory regulation on the marketing of food products, as the most effective way to reduce high fat, salt and sugar foods being marketed to children and other groups’ [53].

In 2016, the UN Special Rapporteur on the Right to Health called on Member States to adopt laws that limit the marketing of unhealthy food and beverages in school-based sporting activities and at professional sporting events. He also stated that Member States should ‘ban the advertising, promotion and sponsorship of all children’s sporting events, and other sporting events which could be attended by children, by manufacturers of alcohol, tobacco and unhealthy foods’ [54].

In line with other legal analyses of this area [12, 58–61], this review shows Article 24 of the UNCRC has been interpreted by subsequent instruments to encompass reducing food and beverage marketing by adopting statutory regulations. All six Category B interpretive instruments mention the need to regulate food and beverage marketing and two mention the inappropriateness of industry involvement through self-regulatory measures. Given that the UNCRC states that Member States have the obligation to take “all appropriate legislative, administrative, and other measures for the implementation” of all rights, the human rights global health law instruments can provide a strong directive to Member States to act.

## Discussion

A wide range of global health and international human rights legal instruments have been used over time by the four UN agencies: seven by the WHO over thirteen years; two by the FAO over one year; three by the UNGA over seven years; and eight by the OHCHR over twenty-one years. The language calling for action on restricting marketing of unhealthy food to children by the WHO, FAO and UNGA was weaker overall than the rights-based instruments from the human rights bodies. The WHO, FAO and UNGA instruments do not explicitly call for Member States to implement government-led mandatory regulations. The strength of the language is also not consistent, does not get stronger over time and varies based on the type of instrument. In contrast, the UN Human rights instruments call for government regulations to be enacted in a more directive manner. All the WHO, FAO and UNGA instruments are ‘soft’ laws and are classified as Category C instruments. There is one Category A binding convention in the human rights field, the UNCRC, and six interpretive instruments (Category B).

Once the WHO Set of Recommendations were introduced the most common language was to call on Member States to implement those Recommendations with varying degrees of strength. The strongest language used by these actors is the WHO Commission on Ending Childhood Obesity in the Resolution that passed the Commission’s final report ‘urging’ Member States to implement the WHO Set of Recommendations. However, after 2016, the language used weakens again. This is exemplified by the 2018 Political Declaration that is the first UN High Level Meeting on NCDs where the WHO Set of Recommendations is not mentioned. Instead, it invites the private sector to commit to further reduce the exposure of children to unhealthy food and beverage marketing.

Of most importance is that the WHO Set of Recommendations does not stipulate what is considered ‘implementing’ in the sense that an industry self-regulatory initiative backed by government could suffice. However, industry initiatives have been shown to be less impactful to reduce the exposure of children to unhealthy food and beverage marketing by a wealth of empirical studies [27–30, 36, 62–66]. Given the evidence that has emerged since the WHO Set of Recommendations were published, that mandatory responses to marketing are more effective than self-regulatory responses [26–29, 35, 62–66] this relaxed directive to Member States is becoming obsolete.

In comparison, the language used by the global actors in the human rights space is stronger. In this sector, there is an overarching convention, the strongest global health law instrument the UN system can implement. While the UNCRC itself is silent on food marketing, the interpretation of the Convention, through the rights-based infrastructure (General Comments, Special Rapporteur reports and Committee reports) and the rhetoric of the UN actors regarding child rights and marketing of unhealthy food includes strong language to encourage or even require government action. Of interest is the language that directly calls on Member States to use legal instruments to regulate the issue. All six Category B interpretive instruments mention the need to regulate food and beverage marketing and two mention the inappropriateness of industry involvement through self-regulatory measures.

While global health law on restricting children’s exposure to food marketing might be inconsistent, the solution is not necessarily to establish a more binding ‘hard’ law instrument like a convention. Global health law has emerged to address a need for a variety of binding and non-binding instruments to deal with the global nature of health issues where traditional international health law instruments, namely conventions, are no longer agile enough to address pressing issues. While the ‘soft’ instruments may appear weaker and therefore less effective, without the flexibility provided by global health law, such instruments may not have been introduced [14, 15, 67]. Removing the onus for state ratification, the soft instruments can help build a negotiated but shared vision for health issues that can codify a new health norm [14, 18].

When the global health law instruments in this study are assessed concurrently, a much stronger picture emerges of the international norm that Member States have agreed to in a multitude of ways through the mechanisms of four different UN actors. The research shows there has been a developing understanding and focus on the issue of restricting unhealthy foods and beverage marketing to children over time. This existing infrastructure could be built upon to further embed the policy action required from Member States.

Other global health instruments could be added to the infrastructure to clarify the obligations of Member States, particularly around introducing mandatory legislative options as opposed to co-regulatory or self-regulatory approaches. For example, in 2019 the WHO UNICEF Child Rights Commission called on the UN system and Member States to introduce an Optional Protocol to the UNCROC to protect children from an array of harmful commodity marketing including sugar-sweetened beverages, which is now under consideration [68]. Optional Protocols to human rights treaties are treaties in their own right, and are open to signature, accession or ratification by countries who are party to the main treaty. The Optional Protocols includes an inquiry procedure, as well as a complaints procedure. An inquiry procedure enables the Committee to conduct inquiries into serious and systematic abuses of children’s human rights in countries that become State parties to the Optional Protocol. The introduction of this global health law instrument would increase the influence of UN actors over Member States to act. The WHO Set of Recommendations should be updated so that industry self-regulation is no longer an option for Member States to meet their commitments under this instrument.

It is important to consider that global health law is only one area of global health governance that can be used to address a global health issue [17, 67, 69]. Other governance tools include: mobilising financial resources or expertise on a subject area, convening multi-stakeholder events to discuss issues, or introducing accountability mechanisms including more stringent monitoring [13, 62]. As such, global health law cannot be considered in isolation as the only lever available to UN actors. There are other levers and mechanisms by which global health issues can get on the global political agenda, mobilise support and expertise and compel Member States to act. Further research could investigate mechanisms to increase accountability and manage conflicts of interest that could be utilised to support and strengthen the existing global health law infrastructure. For example, researching how the UN’s approach to engaging with the tobacco industry could be adopted in relation to the food and beverage industry in the formation of the global health law instruments.

This research illustrates an existing infrastructure of global health law instruments, which in many instances lack strength, but are nonetheless, in combination, a strong base on which to build upon. O’Cathaoir states that the human rights systems should harness the WHO Set of Recommendations (as they pertain to the control and prevention of NCDs) and in return the WHO should leverage the human rights instruments already in existence [70]. This proposition could be directly applied to the specific case of food marketing. If the existing instruments are strengthened there is potential for the global UN actors to have more influence over the issue of children’s exposure to unhealthy food and beverage marketing.

## Conclusion

When the global health law instruments in this study are assessed in conjunction with each other, a much stronger picture emerges of the international norm that Member States have agreed to in a multitude of ways through the mechanisms of four different UN actors. This study shows there has been a developing understanding and focus on the issue of restricting unhealthy foods and beverage marketing to children over time, particularly in the more recent history when the human rights actors have emerged. While some of the existing global health law instruments are weak, in large part due to the impact of the political pressure from private actors and power dynamics of the multiple global health actors at play, the existing infrastructure could be strengthened to call on more Member States to regulate unhealthy food and beverage marketing. Strengthening the directives in the global health law instruments to clarify Member States’ obligations using both WHO and OHCHR mandates could increase the utility of global health law and the influence of UN actors.

## Electronic supplementary material

Below is the link to the electronic supplementary material.

Supplementary Material 1

## Data Availability

The datasets during and/or analysed during the current study available from the corresponding author on reasonable request.

## Abbreviations

NCDs: Non-communicable diseases
UN: United Nations
WHO: World Health Organization
FAO: Food and Agriculture Organization
OHCHR: UN Office of the High Commissioner for Human Rights
UNGA: United Nations General Assembly
WHO Set of Recommendations: World Health Organization Set of Recommendations on the Marketing of Foods and Non-Alcoholic Beverages to Children
ECHO: WHO Commission on Ending Childhood Obesity
