# Supplementary material for: The utilisation of legal instruments by United Nations actors to restrict the exposure of children to unhealthy food and beverage marketing: a qualitative content analysis of UN instruments
Source: Global Health. 2023 Jun 30;19:45. doi: 10.1186/s12992-023-00939-4 (PMC10314450; doi:10.1186/s12992-023-00939-4)
Supplement: Supplementary file 1 — Supplementary Material 1 [file 12992_2023_939_MOESM1_ESM.docx]

# Online supplementary File

# Search methods undertaken

## Full list of Search terms

| UN OR “United Nations” AND | marketing AND food; marketing AND food AND policy; marketing AND nutrition AND policy; marketing AND nutrition; marketing AND food AND law; advertising AND food; advertising AND food AND policy; advertising AND food AND law |
| --- | --- |
| WHO OR “World Health Organization” AND | marketing AND food; marketing AND food AND policy; marketing AND nutrition AND policy; marketing AND nutrition; marketing AND food AND law; advertising AND food; advertising AND food AND policy; advertising AND food AND law |
| FAO OR “Food and Agriculture Organization” AND | marketing AND food; marketing AND food AND policy; marketing AND nutrition AND policy; marketing AND nutrition; marketing AND food AND law; advertising AND food; advertising AND food AND policy; advertising AND food AND law |
| “Human rights” AND | marketing AND food; marketing AND food AND policy; marketing AND nutrition AND policy; marketing AND nutrition; marketing AND food AND law; advertising AND food; advertising AND food AND policy; advertising AND food AND law |
| “Child rights” AND | marketing AND food; marketing AND food AND policy; marketing AND nutrition AND policy; marketing AND nutrition; marketing AND food AND law; advertising AND food; advertising AND food AND policy; advertising AND food AND law |

## Sites searched

United Nations website; United Nations Human Rights Officer of the High Commissioner; the United Nations International Children’s Emergency Fund website; United Nations Digital Library website; United Nations Official Document System; World Health Organization Regional Offices and Headquarters websites; Food and Agriculture Organisation headquarters; Google searches for key terms.

## When searches were conducted

Searches were conducted from April 2021 to April 2022 to ensure any newly adopted or published documents were caught.

## Inclusion and exclusion criteria

Inclusion criteria:

1. The document was a UN level agency instrument that called on Member States to act in some way to reduce children’s exposure to unhealthy food and beverage marketing, as opposed to technical advice to aid Member States such as WHO reports on policy design.
2. To qualify as an ‘instrument’, the document had to be a normative or legal global health law instrument that fit under the governance structures of that entity. A definition of ‘instrument’ was adopted from Moon’s work as ‘a codified rule (whether binding or non-binding) with the explicitly-stated intention to protect or promote health, endorsed by a governmental or intergovernmental entity, agreed by three or more countries and with effects beyond a single region.’
3. The instrument had to be relevant to all Member States, not specific regions. This third criteria was also chosen because the focus of the study was global, so instruments had to address all Member States.

Exclusion criteria:

1. A document that didn’t call on Member States to act in any way. For example, technical advice or expert reports were not included as they were not an instrument agreed on by multiple states and didn’t have the appropriate directive language to assess.
2. Regional instruments were excluded, for example a WHO Regional instrument that otherwise fit the inclusion criteria.
3. A document that was not authored or published by a UN agency.

## Who made decisions about relevant vs irrelevant material

FS made the initial decisions about relevant vs irrelevant material and then all authors reviewed the list of instruments and discussed the final list included in the study.
